# Correlating Movement Impairments As Potential Risk Factors for Musculoskeletal Dysfunction: A Retrospective Cross-Sectional Analysis in a Rehabilitation Setting

**DOI:** 10.7759/cureus.79841

**Published:** 2025-02-28

**Authors:** Deepak Sebastian, Priti George

**Affiliations:** 1 Rehabilitation, Henry Ford Medical Group, Plymouth, USA; 2 Physical Therapy, Institute of Therapeutic Sciences, Plymouth, USA; 3 Physical Therapy, Henry Ford Health, Plymouth, USA

**Keywords:** correlation, cross-sectional, dysfunction, motion impairment, musculoskeletal, prevention, wellness

## Abstract

Background: Mechanical musculoskeletal disorders diminish quality of life and increase healthcare costs but lack prevention strategies. The lack of validated risk factors may be a reason. This study supports the previously hypothesized motion impairments as one potential risk factor in causing mechanical musculoskeletal disorders.

Methods: A retrospective chart review of individuals in a rehabilitation setting with mechanical musculoskeletal disorders was done to identify the presence of co-existing motion impairments. A correlational analysis of their co-existing presence with a description of how they may directly contribute to those disorders followed.

Results: All individuals with mechanical musculoskeletal disorders whose charts were reviewed, presented with co-existing motion impairments. Pearson's correlation coefficient (R) was 1 indicating a strong positive correlation between variables. Linear regression revealed a coefficient of determination (R2) of 1 suggesting that variation in the dependent variable was explained by the independent variable.

Conclusion: The results of this study may be of value as a hypothesis generator highlighting the need for the investigation of motion impairments as a potential risk factor for musculoskeletal dysfunction.

## Introduction

Wellness interventions include screening and testing individuals at appropriately timed intervals to investigate risk and early signs of disease. They also include counseling healthy individuals on lifestyle factors to prevent disease [1,2]. Some aspects of human health receive greater preventive attention than others, such as acute and chronic illnesses that cause death or disability [3]. One aspect of human health however that receives the least structured attention as far as prevention is concerned is musculoskeletal health [4]. While acute, metabolic, and inflammatory musculoskeletal disorders receive some attention, sub-acute and chronic mechanical degenerative conditions receive little or no attention [5]. In many instances, degenerative musculoskeletal disorders are treated symptomatically and waited on till anatomical disruption has occurred for surgical management to intervene. The extended pain management in between appears to have an added financial consequence [6].

In the area of mechanical degenerative musculoskeletal disorders, osteoarthritis (OA) has garnered attention and is directly linked with joint pain. Clinicians are vigilant about identifying advanced OA or cartilage wear, for both medical and surgical indications. In many cases, however, these advanced osteoarthritic changes are not seen in the presence of pain and dysfunction [7]. Clinicians have therefore concluded that it is not just the intra-articular structures (cartilage and subchondral bone) that cause pain, but also the extrinsic extra-articular structures [8]. When the initial diagnostic process does not reveal major joint degenerative changes, the extraarticular structures like the muscle, tendon, bursa, ligament, and nerve, are thought to be initiating pain secondary to stress from mechanical faults [8]. These mechanical faults, also known as "motion impairments," when combined with activity and overuse, are associated with causing painful extraarticular conditions like myositis, tendonitis, bursitis, ligament strains, and nerve entrapments [9-11]. The point being made here is that, besides disease processes like rheumatological, infective, and autoimmune disorders, initiating joint degenerative processes directly, without affecting the extraarticular structures might not be possible [12]. Since they are the first line of defense for ground reaction forces during activity, pain in these structures prior to advanced degeneration or anatomical disruption (with the accompanying presence of motion impairments) can offer clinicians a clue directed toward prevention [12].

Hence, in the management of mechanical musculoskeletal disorders, a suggested requirement may be as follows. When an individual presents to primary care with mechanical musculoskeletal symptoms and investigations do not show advanced degenerative or soft tissue changes, the recommended initial management besides symptomatic interventions may be to perform a prevention screen. The goal is to identify risk factors that may cause a progression of the symptom in question to a more disabling musculoskeletal disorder. However, to effectively execute this process, the development of a structured screening process with validated risk predictors might be necessary [13]. Additionally, it may also be necessary to enlighten clinicians as to how these risk factors can advance a minor musculoskeletal symptom to a more disabling musculoskeletal disorder. This study highlights the previously described motion impairments as one such potential risk factor in contributing to the progression of mechanical musculoskeletal disorders. Their correlative presence alongside conventional musculoskeletal disorders is highlighted with a description of how they contribute to progression. Basic screening tests to identify some of the most common and easily identifiable motion impairments are described with a focus on the lower extremities.

Risk (motion impairment) identification in musculoskeletal practice

Motion impairments at a joint level present as structural asymmetries, and deviations in mobility and strength from the established normal [9-11,14]. They are both obvious as well as subtle, as the examined motion segment may otherwise appear normal, unlike an obvious structural anomaly, break, tear, or dislocation. They may be elicited by passively examining a motion segment [15] or by visualizing structures during postures, movement, or activity [14]. In many cases, a macroscopically normal movement can occur as a compensated activity to overcome an asymmetry. The ability to elicit these compensated movements may require skill and experience. These compensated movements often occur at the expense or overuse of structures that compensate for the asymmetry or impairment rendering them vulnerable to injury and dysfunction [16,17]. A symptomatic approach without addressing persistent motion segment impairments may eventually lead to common degenerative and anatomically disruptive musculoskeletal disorders seen in clinical practice. An ideal management strategy may be to focus on the identification and management of these motion impairments at the very beginning in combination with the standard symptomatic approaches [18-20].

It is important to comprehend the difference between clinical tests that identify a musculoskeletal disorder versus impairment-based tests that recognize joint and motion segment dysfunction that may eventually lead to a musculoskeletal disorder [21]. An analogy would be a clinical test for a myocardial infarct (cardiac enzymes) versus clinical tests to identify a brewing dysfunctional process or risk factors for heart disease (high cholesterol, hypertension, and obesity) that when left unmanaged would possibly or eventually lead to a myocardial infarct. Similarly, in an individual presenting with minimal signs of musculoskeletal dysfunction, clinical tests may help recognize the presence of tendinitis, bursitis, ligament sprain, or neuritis. The addition of a musculoskeletal screen exam incorporating motion impairment testing may reveal the treatable causes for vulnerability and susceptibility to these conditions [9,11,14].

As the list of motion impairments is elaborate, the most common and more easily identified motion impairments seen in lower extremity mechanical musculoskeletal dysfunction are listed. Their relationship to a broad range of conventional degenerative or overuse musculoskeletal diagnoses, commonly seen in musculoskeletal practice is also described. Another interesting feature that is highlighted is the regional interdependence where pain in the knee could arise from a motion impairment in the hip and pain in the hip could arise from a motion impairment in the ankle [22,23]. The common and more easily identifiable motion impairments are as follows: (i) hip: iliopsoas and hip capsule tightness, gluteus maximus, and gluteus medius weakness; (ii) knee: knee extension lag; and (iii) ankle/foot: lack of pronation and supination reversal.

## Materials and methods

Study purpose

The aim of this study was to investigate the correlation between mechanical lower extremity musculoskeletal dysfunction and co-existing motion impairments. The goal was to identify a potential risk factor that when appropriately managed, may aid in early prevention and progression.

Ethics statement

Approval was obtained for this study from the Institutional Review Board (IRB) of the Henry Ford Health System, in Detroit, Michigan as a non-human subject research (non-HSR) project. The study incorporated a retrospective chart review and hence no human subjects were (directly) involved in the data collection.

Study population and sample size

A retrospective investigation of the charts of all patients having attended a hospital-based outpatient physical therapy clinic with lower extremity mechanical pain and dysfunction for a six-month period (January 1, 2024, to June 30, 2024) was performed. The patients were from the schedule of one board-certified orthopedic physical therapist experienced in assessing motion impairments. However, two clinicians board-certified in orthopedic physical therapy with over 20 years of clinical experience in utilizing movement impairment assessments participated in the investigation. Prior to the investigation, both clinicians agreed upon the inclusion and exclusion criteria and the list of co-existing motion impairments to be identified. In the six-month period, 28 patients met the established inclusion criteria and hence a total of 28 charts were reviewed (n=28) consecutively.

Study design

The inclusion criteria were mechanical pain in the hip, knee, ankle, and foot. The exclusion criteria were inflammatory or metabolic joint disorders of the lower extremity, joint replacements, current joint, tendon, ligament, or other joint and soft tissue surgeries. A number was assigned to each regional dysfunction as follows, hip-one, knee-two, ankle, and ankle foot-three. During the retrospective chart review the presence of motion impairments (identified during initial evaluation) co-existing with the regional pain and dysfunction were identified, a process routinely done in all standard physical therapy evaluations. Three sections were created in the data table (Table 1); regional pain and dysfunction, the presence of at least two related motion impairments, and the absence of related motion impairments. In the presence of at least two related motion impairments, the same number was assigned in the motion impairment section in the data table as assigned to the regional pain and dysfunction section of the data table. For example, if an individual presented with hip pain and trochanteric bursitis and presented with hip capsule tightness and gluteus medius weakness, the motion impairment section was also assigned number one. The absence of related motion impairments received an assignment of zero. If the numbers assigned to the regional pain and dysfunction section and motion impairment section were the same, then the indication was that there were at least two causative, coexisting motion impairments present during the initial screening and evaluation process.

**Table 1:** Table of dysfunctions and associated impairments.

Regional dysfunction	Coexisting impairments	Absence of impairments
Hip-1 Knee-2 Ankle & Foot-3 (n=28)	1, 2, 3 (n=28)	0 (n=28)
1,2,2,1,2,2,2,1,2,1,1,3,2,1.	1,2,2,1,2,2,2,1,2,1,1,3,2,1.	None.
2,2,2,2,2,2, 2,1,2,3,3,2,1,3.	2,2,2,2,2,2, 2,1,2,3,3,2,1,3.	

Study measures

A correlation analysis was done between a presenting lower extremity musculoskeletal dysfunction and the presence of causative, co-existing motion impairment, alongside. The objective was to justify that when the hip, knee, or ankle exhibited pain and dysfunction, the above-mentioned motion impairments indeed co-existed, supporting the hypothesis that they might have had a part in causing the dysfunction. The higher the correlation of co-existence the better the justification that the listed motion impairments may have contributed to the diagnosis. Following the correlational justification of the coexistence of motion impairments, the physiological basis of how these motion impairments might have contributed to causing the diagnosis is described.

Statistical analysis

Pearson’s correlation coefficient (R) was used to investigate the correlation between regional pain and dysfunction (independent variable X) and co-existing motion impairments (dependent variable Y). Linear regression was done to predict the value of the dependent variable based on the value of the independent variable. The coefficient of determination (R2) was used to investigate if the variation in the dependent variable (Y) was explained by the independent variable (X), in the regression model.

## Results

All 28 patients investigated with regional pain and dysfunction in the lower extremity showed at least two related co-existing motion impairments. Pearson’s correlation coefficient and linear regression were used to analyze relationships between X and Y [24] where X was the regional diagnosis and Y was the coexisting motion impairment(s). Pearson’s correlation coefficient (r) was 1 indicating a strong positive correlation between independent variable (X) and dependent variable (Y). Linear regression was done to predict the value of the dependent variable based on the value of the independent variable. The coefficient of determination (R Square) was 1 suggesting that 100.00% of the variation in the dependent variable (Y) was explained by the independent variable (X) included in the regression model (Table 2).

**Table 2:** Data analysis.

Pearson's correlation	Linear regression
R calculation and summary	R Square calculation and summary
r = ∑((X - M_y_)(Y - M_x_)) / √((SS_x_)(SS_y_))	ŷ = bX + a
r = 11.429 / √((11.429)(11.429)) = 1	b = SP/SS_X_ = 11.43/11.43 = 1
r = 1	a = M_Y_ - bM_X_ = 1.86 - (1*1.86) = 0
Correlation:1	ŷ = 1X + 0
	Regression Equation: y^= 0 + 1x_1_
	R Square:1

**Figure 1:**
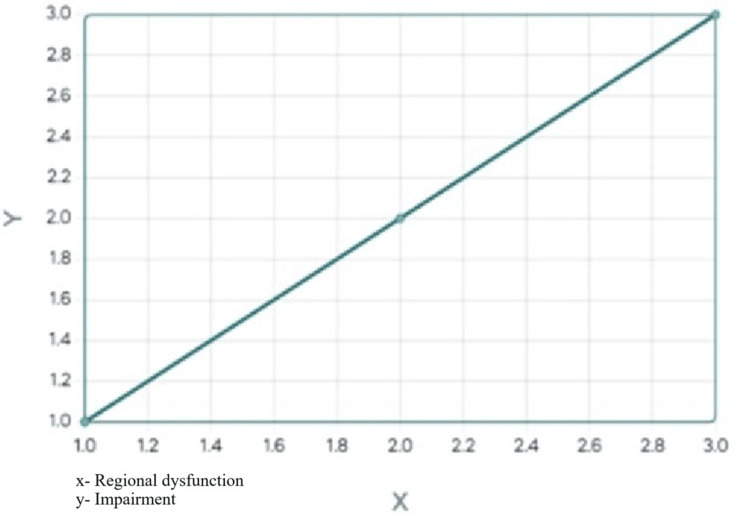
Scatter plot result with line of best fit.

The slope of the regression line equaled 1 suggesting that for every one unit increase in the independent variable (X), the dependent variable (Y) was also expected to increase by 1 unit (Figure 1). The correlational analysis was to statistically highlight the consistent presence of motion impairments, if any, along with musculoskeletal disorders. The results of the correlational analysis statistically support and highlight the co-existence of motion impairments(s) alongside the musculoskeletal disorders in the population investigated.

## Discussion

The literature review in this section highlights how unmanaged motion impairments combined with activity and overuse can lead to established mechanical musculoskeletal disorders. It is not all-encompassing, but the most obvious and validated motion impairments seen in lower extremity musculoskeletal dysfunction are described.

Motion impairment in the hip

While a multitude of differentials exists for musculoskeletal hip pain the commonest seen in primary care are osteoarthritis and impingement presenting as groin pain, tendinitis of the glutei and trochanteric bursitis presenting as lateral hip pain, lumbopelvic dysfunction and piriformis syndrome presenting as posterior hip pain [25]. In the absence of trauma, a middle-aged or older individual presenting with hip pain may tend to first have a clinical evaluation focused on ruling out non-musculoskeletal causes ranging from vascular, inflammatory, infective, neoplastic, deficiency, drug-mediated, congenital, autoimmune, and endocrine disorders. While a smaller proportion of individuals present with these sources [26], including advanced osteoarthritis requiring an orthopedist for a potential joint replacement, the larger majority receive a label of early arthritis or extraarticular soft tissue strains and are treated symptomatically. While this is a good start, the preventive aspect is lost when the initial management concludes with symptomatic therapies without the investigation and management of motion impairments. If so, the potential for recurring pain and dysfunction is highly likely [14]. A description of the most common motion impairments in the hip is as follows.

Tightness of the hip flexors and hip capsule

The iliacus and psoas muscle groups work to accomplish hip flexion which is an important functional activity to initiate a forward motion of the leg [27]. Owing to their extent from the lumbopelvic region to the upper part of the femur, any functional position that brings the femur closer to the pelvis anteriorly (sitting), shortens these muscle groups. Hence it is common to see tightness of the hip flexors in middle-aged adults due to extended periods of sitting [28].

The hip capsule and blended ligaments maintain and control functional mobility and stability. The orientation of the capsule is such that it is oblique and runs superolateral to inferomedial. In a functional position where the knees are kept apart the posterior fibers shorten and vice versa with the knees together the anterior capsule shortens. The shortening when prolonged (prolonged sitting especially cross-legged or legs apart) can lead to tightening resulting in the characteristic FABER (flexion, abduction, external rotation) and FADIR (flexion, adduction, internal rotation) presentation [29].

Weakness of the glutei

A fully relaxed hip flexor group and anterior hip capsule are important for the optimal functioning of the much-needed hip extensor group (gluteus maximus and hamstrings) on the opposite side [30]. The efficiency of the gluteus maximus is diminished with chronically tight hip flexors due to lack of range and excursion [30]. Additionally, sedentary lifestyles can also predispose to deconditioning [31]. The other important muscle group the gluteus medius, requiring a neutral frontal plane to function optimally, is relatively diminished in efficiency as the tight hip flexors move the femur (and thigh) relatively forward from the frontal plane [32].

Clinical implications of motion impairment in the hip (mechanical hip discord)

A musculoskeletal clinician treating mechanical hip dysfunction may encounter the above-described mechanical challenges to occur collectively. An explanation of how this collective discord (hip capsule and hip flexor tightness, glutei weakness) can cause the commonly observed mechanical musculoskeletal disorders of the hip [33-36] is as follows.

Prolonged sitting has unfavorable consequences on the hip capsule and the hip flexor muscles [37] and depending on the leg position (legs apart or legs crossed) the posterior or anterior capsule may shorten respectively (FADIR and FABER) [37,38]. Movement in the hip comprises movement of the femoral head over the acetabulum, and during gait, the undesirable tightness may create stressed and compressed rotation with the initiation of a friction cascade [39-41]. It is speculated that this increased demand between the femoral head and the acetabulum is one contributor to the initiation of the wear and tear process of degenerative osteoarthritis.

Rotation is described to be an important component of the static and dynamic activity of the hip [39]. Additionally, a balance of the internal (gluteus medius, gluteus minimus) and external rotators (piriformis) of the hip is described as a requirement to maintain an optimal position of the greater trochanter [39,42]. An imbalance of the co-contraction strength of the rotators of the hip secondary to weakness or co-existing hip capsule tightness can alter the position of the greater trochanter increasing the vulnerability of the structures that attach or cross over it. This includes the gluteus medius and minimus tendons, adjacent bursa, gluteus maximus, and iliotibial band (ITB). With an altered position, these structures may be vulnerable to the demands of the altered mechanics when combined with activity and overuse. The resulting pain and dysfunction may present as gluteus minimus tendinopathy, trochanteric bursitis, and ITB friction.

The commonly seen hip flexor tightness diminishes the normal excursion of the gluteus maximus and hamstrings thereby decreasing their efficiency [28]. Propulsive activity as in walking and climbing stairs and transfer activity as in rising from the floor or from sitting is accomplished by an optimally functioning hip extensor group [39,40]. The presence of tight hip flexor muscles accompanied by inhibited or weak hip extensor muscles can create challenging demands on the performance of these much-needed functional tasks. Paradoxically it is common to see tightness of the hip flexors in middle-aged adults and one potential described cause is extended periods of sitting [28].

The gluteus medius requiring a neutral frontal plane to function is challenged in the presence of tight hip flexors. This tightness favors the movement of the femur (and thigh) forward relative to the frontal plane [32]. While the ability of the gluteus medius is diminished in this plane the deep external rotators (piriformis) and the tensor fascia lata (TFL) perform compensated abduction (as it is critical during walking). When prolonged because of overuse, pain, and dysfunction may result in the form of gluteal tendinopathy, TFL strain, and piriformis syndrome [42].

The gluteus medius functions to abduct the hip and stabilize the pelvis during a one-legged stance. Weakness of the gluteus medius results in a downward dip of the pelvis on the opposite side and a compensating lurch on the same side, during a one-legged stance [43]. This contralateral dipping and ipsilateral lurch can potentially irritate the trochanteric bursa by increasing the tension of the ITB and glutei over the bursa, resulting in trochanteric bursitis [35]. Additionally, the contralateral pelvic dip can alter greater trochanteric positioning resulting in ITB tension and friction [44].

A chronically shortened hip flexor group has been related to anterior tilting of the pelvis with increased lumbar lordosis [45,46]. When it is prolonged and combined with lurching from the resulting gluteus medius weakness, the lumbar facet joints and sacroiliac joints may be stressed resulting in lumbar radiculopathy and sacroiliac joint pain [33,34,36,43].

The gluteus maximus and hamstrings work conjointly to extend the hip for functional tasks. An ideally stronger gluteus maximus exerts synergistic dominance over the hamstrings, which reverses when the gluteus maximus is inhibited and weak. This reversed synergistic dominance is typically indicated by frequent cramping of the hamstring muscle with potential hamstring tears when demanded in excess [46].

Common conditions associated with motion impairment of the hip

Early osteoarthritis, hip impingement, trochanteric bursitis, iliotibial band friction, hamstring strains and tears, gluteus medius, gluteus minimus, and tensor fascia lata strain, piriformis syndrome, lumbar and sacroiliac dysfunction.

Motion impairment screen exam of the hip

Examining FADIR

**Figure 2:**
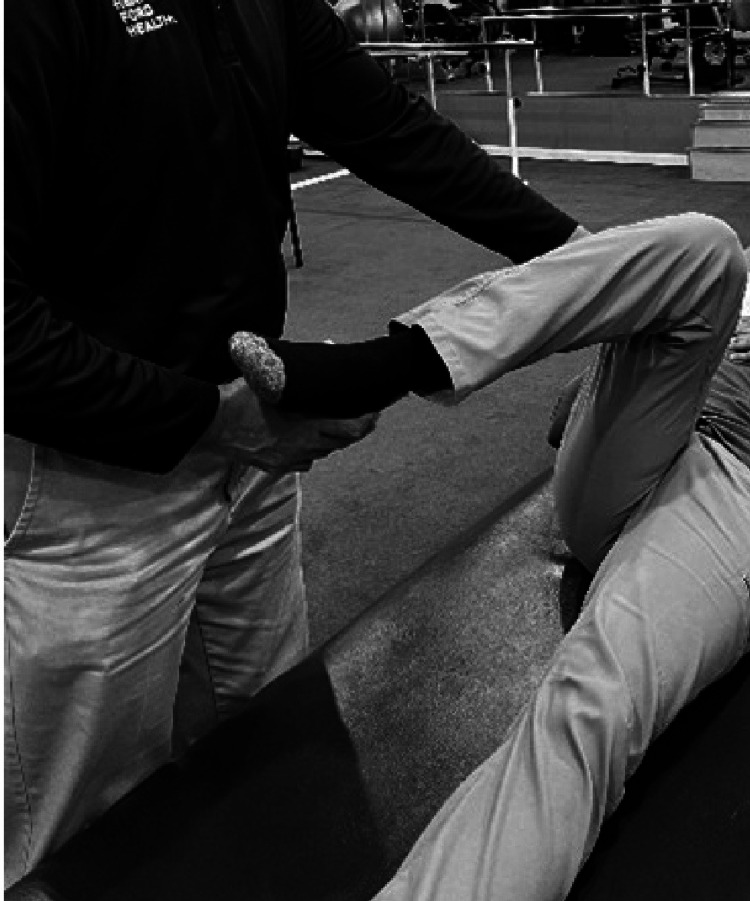
Examining FADIR. FADIR: Flexion, adduction, internal rotation.

The patient is placed supine, and the hip is then passively flexed, adducted, and internally rotated to assess for mobility (normal 45 degrees). The examiner is cautioned to never do this on a hip that has undergone arthroplasty or is unstable (Figure 2). The range is compared with the other side [47].

Examining FABER

**Figure 3:**
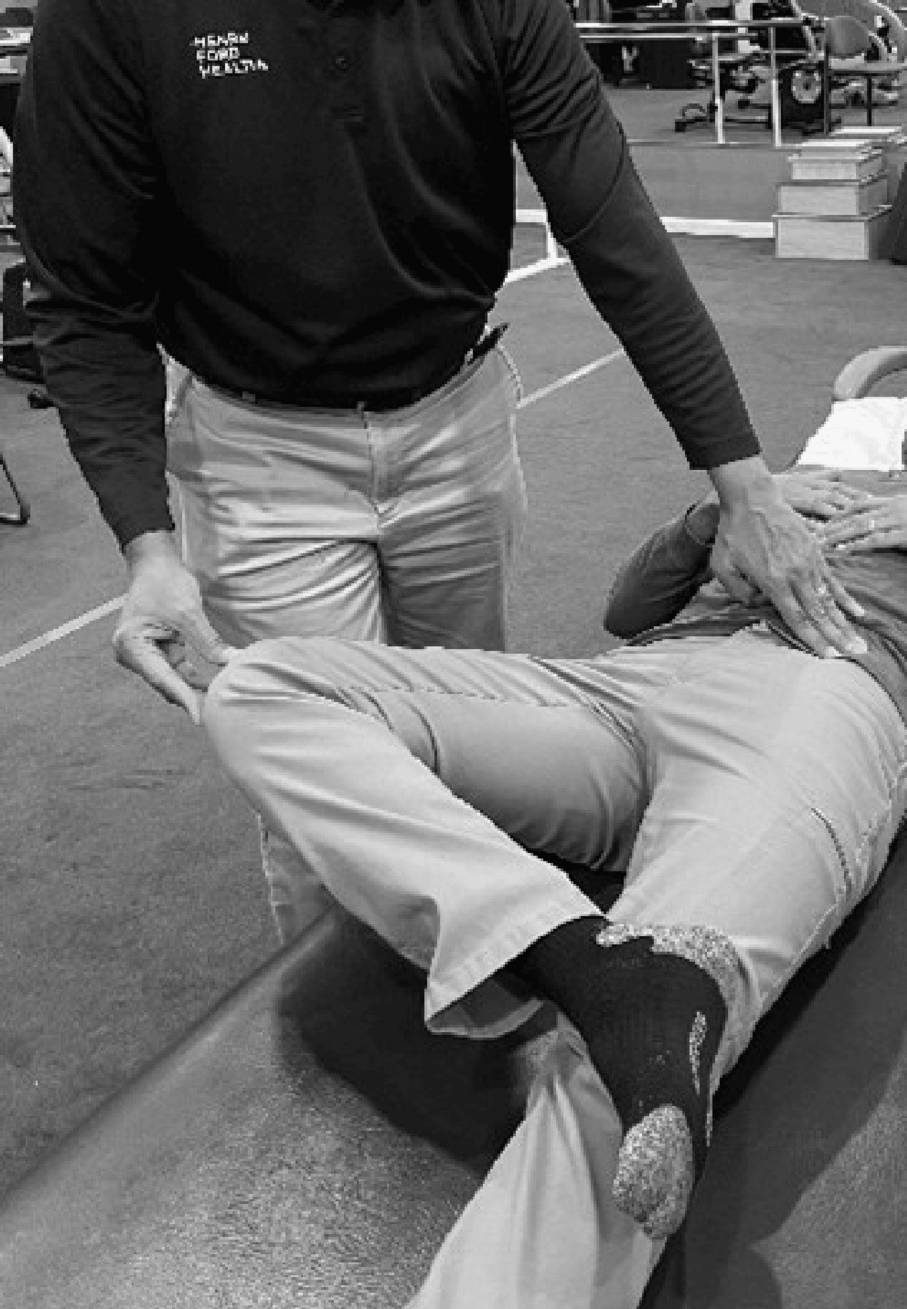
Examining FABER. FABER: Flexion, abduction, external rotation.

The hip is then abducted and externally rotated while maintaining flexion to assess for mobility (normal 50 degrees) (Figure 3). The range is compared with the other side [47].

Examining Hip Flexor Tightness

**Figure 4:**
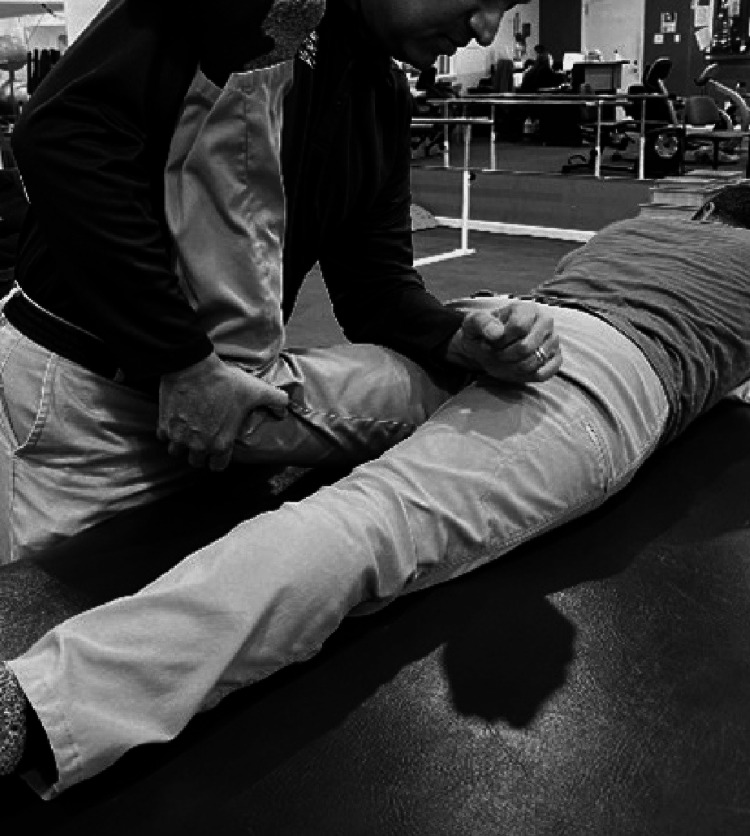
Examining hip flexor tightness.

While the Thomas test is the test of choice to assess hip flexor tightness [45] it may be a difficult position to assume for many patients [48]. If the prone lying position is achievable the examiner blocks the posterior aspect of the hip just below the gluteus maximums (at the level of the trochanter). This prevents the lumbar spine from extending. The femur is then extended by raising the knee to observe range. The range is compared with the other side (Figure 4). Strength testing in this position is also an indicator of range. The inability to extend the hip in this position may be an indicator of both weakness and tightness [30].

Examining Gluteus Medius Weakness

**Figure 5:**
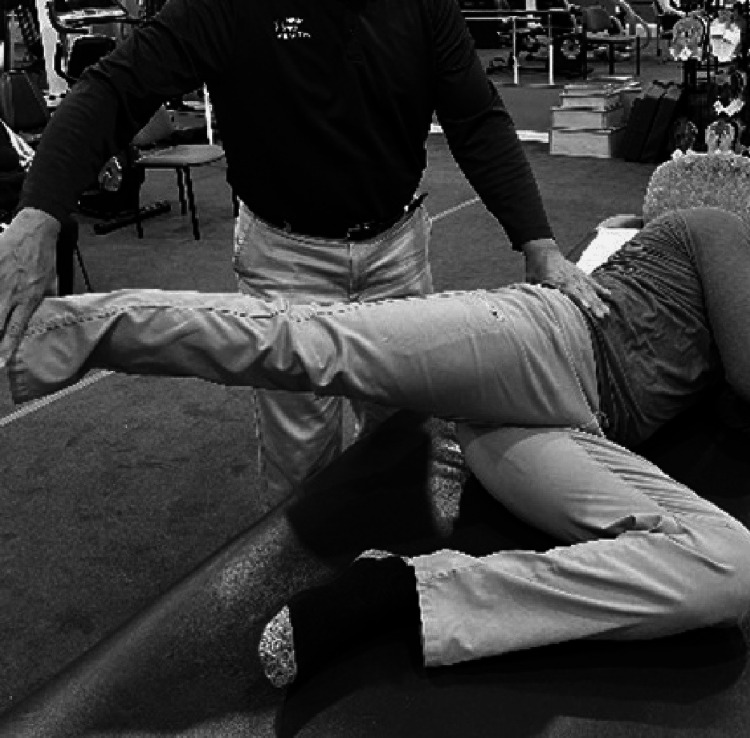
Examining gluteus medius weakness.

With the patient in the side-lying position, the leg is positioned in line with the trunk and not in slight flexion. The pelvis is strictly neutral with the trochanter perpendicular to the ceiling. The hip is rotated internally and resistance to abduction is applied (Figure 5). The inability to maintain resistance may indicate weakness or deconditioning [49].

**Figure 6:**
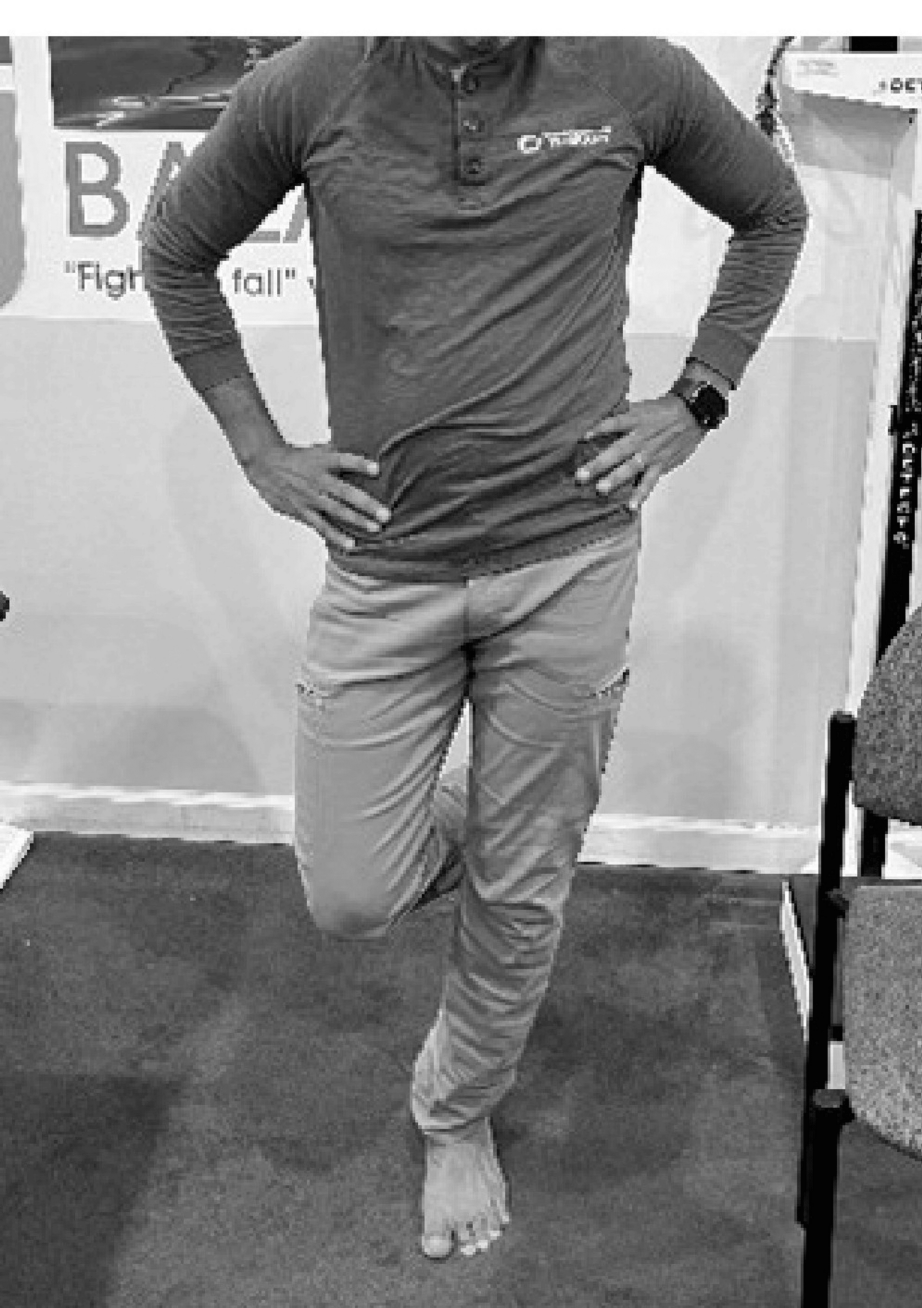
Testing gluteus medius weakness (weak left side).

The single-leg stance is performed by the individual standing with the arms by the sides or the waist while one leg is bent 45 degrees off the floor [50]. The examiner observes for a pelvic drop on the opposite side of the stance leg or a trunk lean to the same side of the stance leg to indicate gluteus medius weakness on the stance side (Figure 6). This test may not be sensitive for the gluteus medius in the presence of balance or neurological disorders.

Examining Gluteus Maximus Weakness

**Figure 7:**
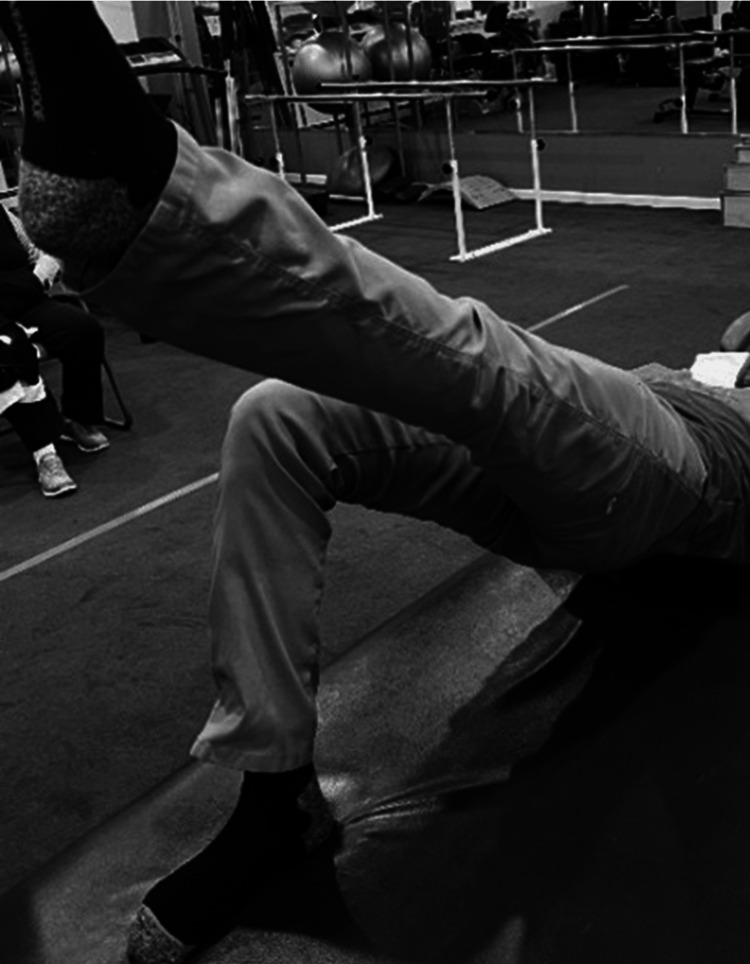
Examining gluteus maximus weakness.

The single-leg bridge is performed in the supine lying position with knees bent and feet supported on the examination table [41,47]. One leg is extended and held or supported while the other leg is used to raise the pelvis off the table until the shoulders, hips, and knees are in a straight line (Figure 7). The inability or difficulty to raise the pelvis off the table may indicate weakness or deconditioning of the gluteus maximus. Occasionally the hamstring cramps to assist with the movement.

Motion impairment in the knee

The knee joint possesses two degrees of freedom, flexion and extension. These two motions while occurring in a straight plane are accompanied by rotation of the femur and tibia [40,51]. Tibial rotation, being an important subcomponent [51] appears to occur in conjunction with femoral rotation in the acetabulum [40]. It is described that maximum internal rotation of the femur occurs in mid-stance stabilized by the gluteus medius which is a hip internal rotator [40]. Tibial external rotation called ‘screw home’ also occurs during stance maintaining a stable knee [51]. It makes sense that femoral and tibial rotations occur in opposition and complement each other during normal mechanics.

Terminal knee extension or screw home, accompanied by tibial external rotation is an important component of the stance phase of gait, for stability [51,52]. Lack of terminal knee extension is often encountered and is a position of relative tibial internal rotation which can be elicited by movement testing [52]. The persistent lack of terminal knee extension, termed an extension lag is described to cause a range of knee dysfunctions seen in practice [52]. The muscles responsible for tibial internal rotation are popliteus, semitendinosus, semimembranosus, sartorius, and gracilis, and external rotation are biceps femoris and vastus lateralis [52]. The assumption that lack of terminal knee extension has rendered the tibia in a position of tibial internal rotation has been described [52-54].

Clinical implications of motion impairment at the knee

Terminal knee extension lag and flexion contractures have been associated with osteoarthritis [55], knee surgery [56,57], arthrogenic muscle inhibition (AMI) [58], gastrocnemius and hamstring tightness [59,60], and knee trauma [61]. The persistence of knee extension lag creates a loss of congruence of the articular surfaces of the tibia, femur, and patella. This can increase tibiofemoral and patellofemoral cartilage surface contact pressure [62,63] leading to degenerative changes when combined with prolonged and progressive loading.

The menisci distribute load and offer stability, lubrication, and proprioception in the knee joint. They are embedded between the femoral and tibial condyles and improve the congruence and stability of the knee joint. In normalcy the tibiofemoral contact area is uniformly distributed, preventing increased demands to the meniscus. In the presence of an extension lag, this uniformity is lost with increased stress and demands on the menisci to distribute loading. A similar change in the contact area with increased stress can also occur following meniscectomy [64]. with additional demands if there is a co-existing extension lag.

The anterior cruciate ligament (ACL) functions to restrain excessive anterior translation and internal rotation of the tibia [65]. Prolonged internal rotation as seen in an extension lag creates a constant pre-stress on the ligament making it vulnerable to injury. Additionally, researchers have described vulnerable peak axial compressive forces that can cause ACL injuries at small knee flexion angles, a position where the tibia is internally rotated [65].

Common conditions associated with motion impairment of the knee

Medial compartment friction (OA), patellofemoral compression, meniscus strain, ACL strain.

Motion impairment screen exam of the knee

Examining Active Lag

**Figure 8:**
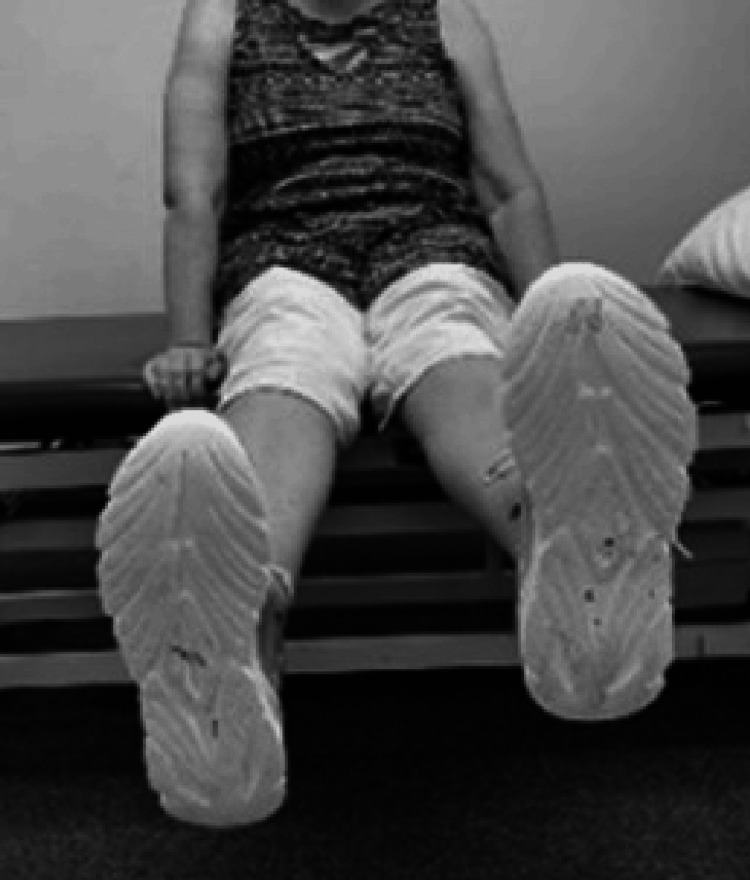
Examining active lag (lag on the right).

In the sitting position with the thighs supported the leg is fully extended to maximum knee extension and the levels of the toes are observed (Figure 8). The lower side may indicate an active knee extension lag [52].

Examining Passive Lag

**Figure 9:**
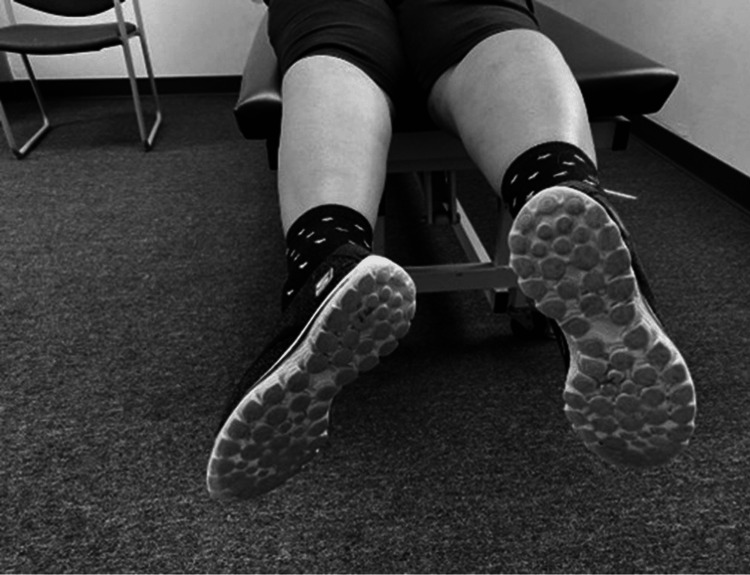
Examining passive lag (lag on the right).

In the prone position with the knees supported over the edge, the leg is fully extended to maximum knee extension, and the levels of the heels are observed (Figure 9). The higher side may indicate a passive knee extension lag [52].

Motion impairment in the ankle and foot

The ankle and foot support and propel the body unilaterally with their complex and flexible articulations assisted by the intrinsic foot and lower leg musculature [66]. Two visible components responsible for the execution of this function in the stance phase of gait are the uninterrupted reversals of pronation (flat foot) and supination (high arch foot) [67]. Pronation is described as a loose-packed position occurring from heel strike to mid and early late stance. It is a position where there is a deformation of the medial longitudinal arch (flat foot) for shock absorption, adaptation on uneven terrain, and maintenance of equilibrium [66]. In the late stance phase, reversal into supination begins with the elevation of the medial longitudinal arch (high arch foot) to enable the foot to transform into a rigid lever for optimal push-off [68]. A pathomechanical situation occurs when this reversal ability is lost due to either a chronically pronated or supinated foot [69]. In a chronically pronated foot propulsion may occur on a pronated loose-packed foot creating tensile overstretching. In a chronically supinated foot, the initial loading may occur on a rigid close packed supinated foot creating shock and buckling. Both scenarios render the foot vulnerable to injury, pain, and dysfunction, during overuse [69,70].

Clinical implications of motion impairment in the ankle and foot

A pronated foot lacking reversal into supination presents with a flattened medial longitudinal arch and the rear foot assuming a valgus position [67,71]. This increases tensile stresses on the plantar medial aspect of the ankle and foot with a lack of a rigid lever for push-off, causing medial and plantar ankle and foot dysfunction including plantar fasciitis and plantar nerve entrapment [67,69,72,73]. The pronation occurring during early stance requires the tendo-achilles to exercise the ability to decelerate the foot into pronation for shock absorption. Persistent pronation or the lack of reversal may challenge the tendo-achilles to decelerate pronation in excess. Additionally, this may challenge and stress the medial and lateral whipping ability of the tendo-achilles resulting in tendinopathies [74,75]. In the presence of persistent pronation, the other decelerators of pronation namely the tibialis posterior and anterior experience increased demands in the effective lowering of the arch during the shock absorption phase of gait. When repetitive they are described as being vulnerable to cause periostitis and medial tibial stress syndrome or "shin splints" [76,77].

A rigid supinated foot presents an elevated medial longitudinal arch and the rear foot assumes a varus position. This increases tensile stresses on the lateral aspect of the ankle causing the ankle to invert during landing [70]. The peroneal muscles function to prevent this inversion and delayed peroneal muscle activation has been described to favor inversion or supination moments, stressing the laterally placed structures [78]. This can range from lateral ligament strains to fractures [79].

Common conditions associated with motion impairment in the ankle and foot

Plantar fasciitis (pronation), lateral ligament strain (supination), achilles tendinosis (pronation), shin splints (pronation), and plantar nerve entrapment (pronation).

Motion impairment screen exam in the ankle and foot

Observing the Rear Foot and Medial Longitudinal Arch

In the standing closed chain position, the examiner observes the rear foot and medial longitudinal arches. A closed chain refers to a motion that occurs in the weight-bearing position and an open chain to that which occurs in the non-weight-bearing position [80].

**Figure 10:**
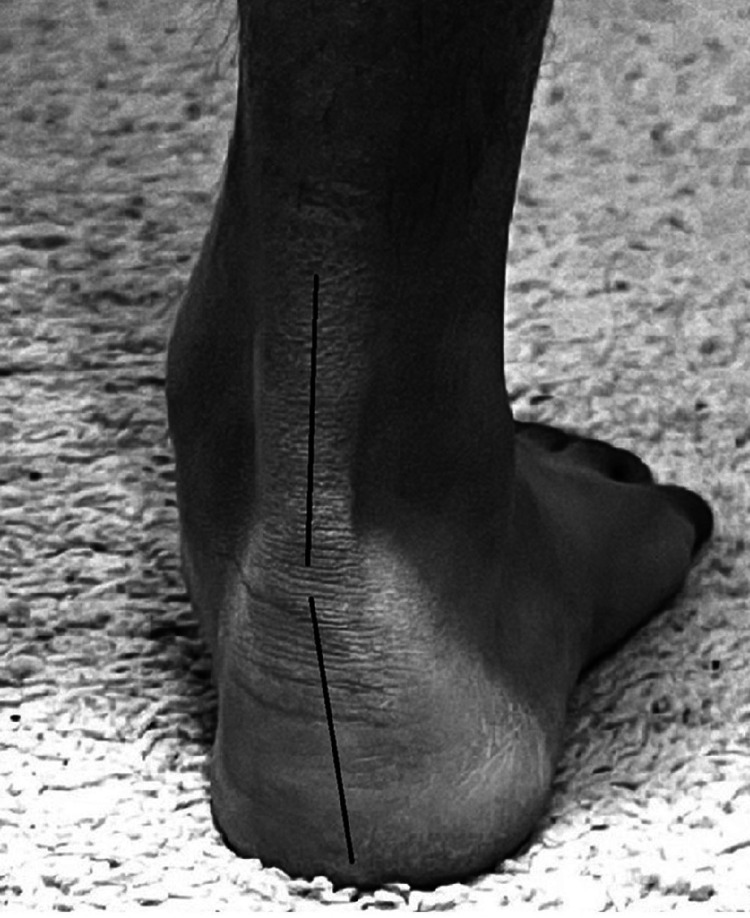
Pronated foot right side.

In a pronated foot [67,71] the rear foot is in valgus with the medial longitudinal arch dropped. The components of a pronated foot begin with the tightness of dorsiflexion in the talocrural joint. Dorsiflexion tightness or the difficulty of performing dorsiflexion and inversion in the closed chain position is characteristic of a pronated foot [67]. The subtalar joint that is immediately below it assumes an everted position (Figure 10) which subsequently drops the medial longitudinal arch along with the mid tarsal joints. A mild inversion twist in the forefoot levels the foot and gives the foot a characteristic "flat foot" appearance.

**Figure 11:**
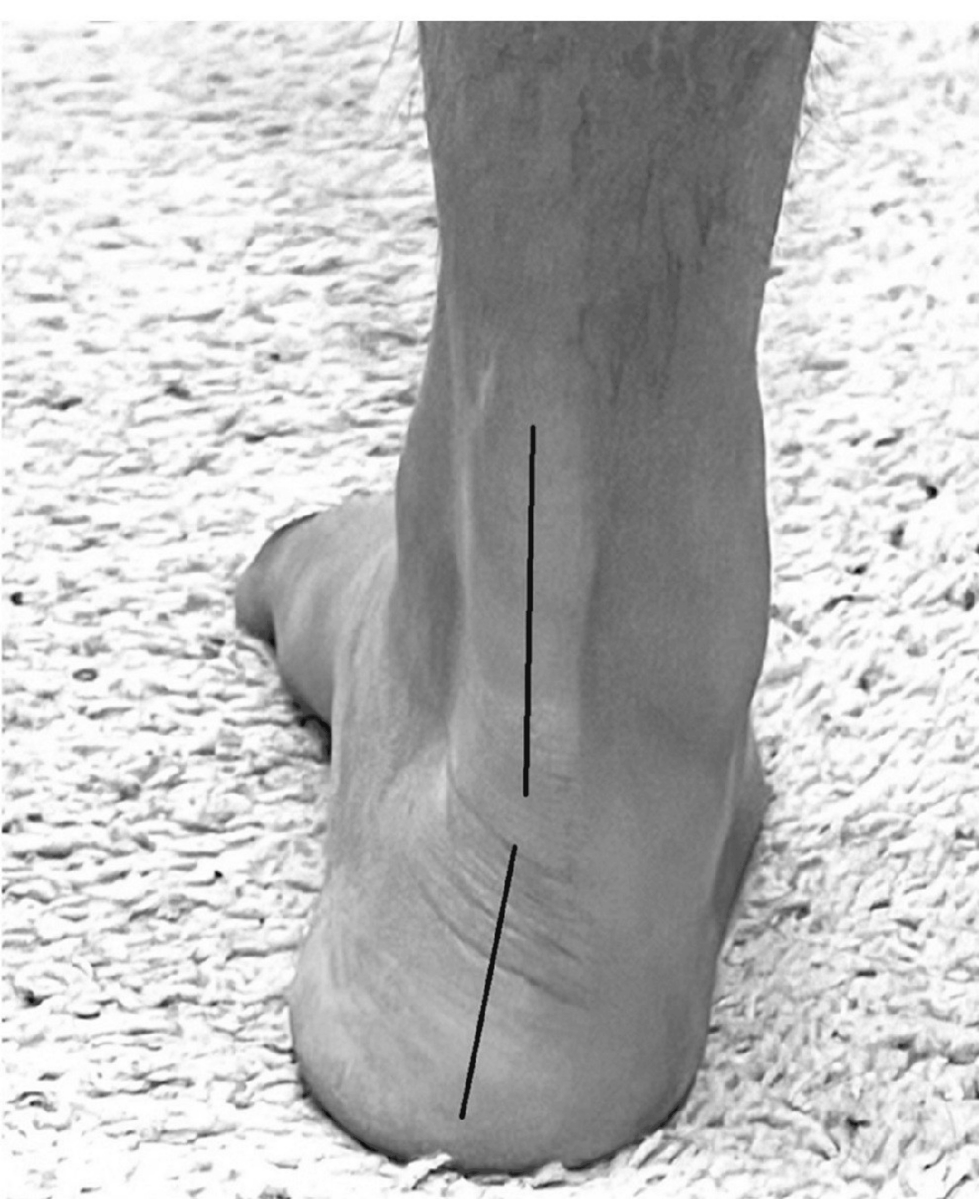
Supinated foot right side.

In a supinated foot, the rear foot is in varus (Figure 11) with the medial longitudinal arch elevated [70,78].

Examining Supination Resistance Test (SRT)

**Figure 12:**
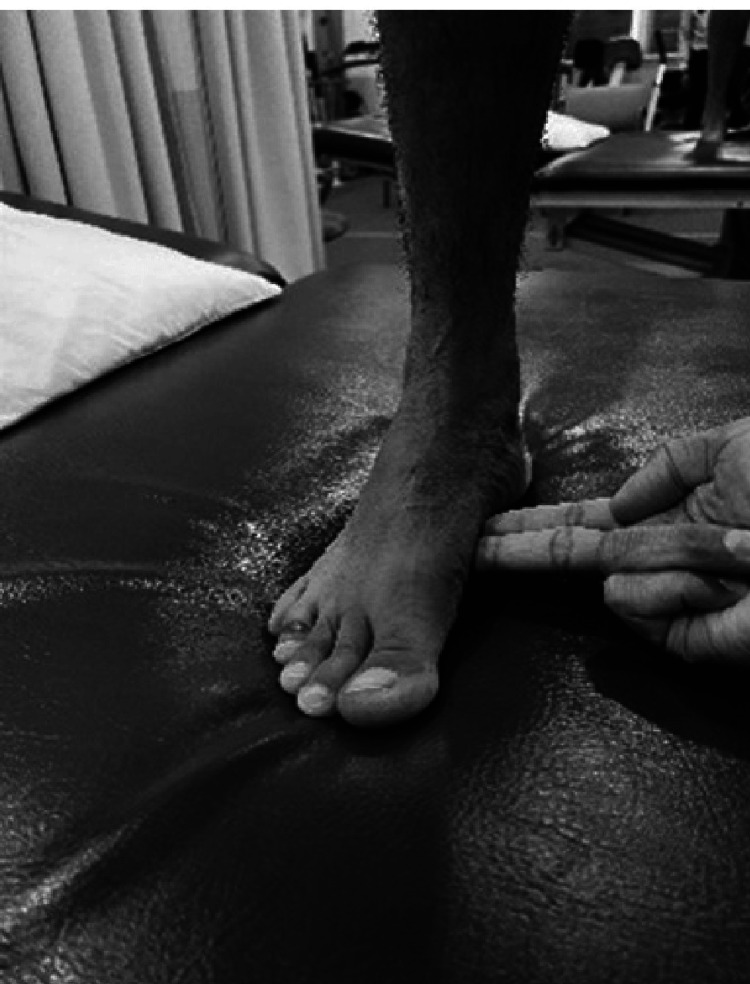
Examining supination resistance test.

This test is performed in the presence of excessive pronation [81]. With the patient standing in relaxed weight bearing the examiner uses two fingers on the medial side under the talonavicular joint. The medial longitudinal arch is elevated by applying a force in an upward direction (Figure 12). The amount of force needed is noted. In a pronated foot, it is harder to elevate the medial longitudinal arch.

Examining Active Supination Dorsiflexion Test (ASDT)

**Figure 13:**
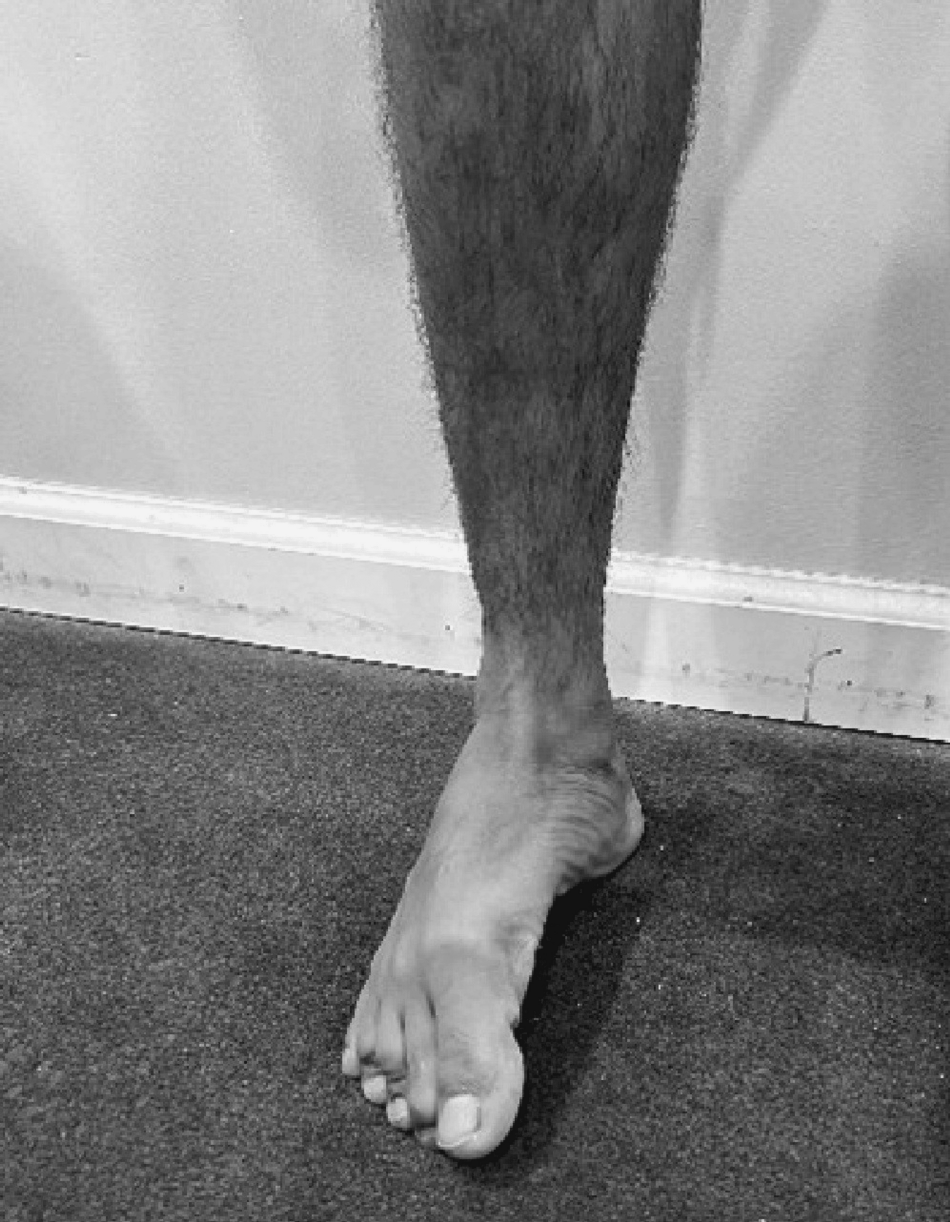
Active supination dorsiflexion test.

This test is performed in the presence of excessive pronation. The patient is made to stand in a comfortable weight-bearing position and asked to curl the toes to raise the arch, turn the foot inward (supinate), and raise the foot upward (dorsiflex) (Figure 13). The movement is compared with the uninvolved side. Typically, the involved side may exhibit a decreased ability to perform this activity [67].

Observing Wear in the Medial and Lateral Heel of Footwear

The examiner can observe for wear on the outer heel of the footwear of the patient. Pronators typically exhibit a wear pattern over the inner heel (medial) and supinator’s outer heel (lateral) [82,83].

Interdependence of the lower extremity kinetic chain

The lower extremity chain comprising the hip, knee, and ankle intricately complement each other to perform functional tasks during weight bearing. A challenge in the optimal functioning of one region directly affects another region within the chain. The literature suggests that abnormal mechanics in the hip including tightness and weakness can affect the normal functioning of the tibio-femoral and patellofemoral articulations with undue stress on the soft tissue structures [84,11]. A similar relationship exists between the hip and the ankle and foot. Studies suggest that hip motion impairment and alterations in muscle performance are related to knee, lower leg, ankle, and foot injuries [85]. Similarly, distal involvement affects the proximally placed knee and hip. The literature describes abnormal mechanics in the ankle and foot directly contributing to knee and hip dysfunction. A pronated foot for example is described to affect rotational stress in the tibiofemoral joint secondary to an alteration in the coupling between the rear foot and tibia. This has been known to increase frictional stresses in the knee joint causing pain and dysfunction [10,23]. Abnormal ankle and foot mechanics have also been described to cause pain and dysfunction in the proximally located hip and pelvis [86].

Study limitations

A perfect correlation between variables may be associated with the small sample size and analysis done by experienced clinicians. Secondly, as the assessment of motion impairments is not common practice, there is limited awareness among clinicians of their intimate association with mechanical musculoskeletal dysfunction. This may render the overfitting correlational presence of motion impairments to appear far-fetched.

Limitations of this study include a lack of standardized assessment protocols for diagnosing motion impairments, observer bias secondary to limited evaluators, and a retrospective design. Given these limitations, while a causal conclusion may not be possible, the results may be of value as a hypothesis generator highlighting the need for investigating motion impairments as a potential risk factor for musculoskeletal dysfunction. Future studies involving a larger population, a prospective design, and utilization of tests with standardized diagnostic utility values performed by multiple investigators, are recommended.

## Conclusions

When regional joint pain and relative dysfunction are the most apparent presentation without major structural changes (yet), it may be of value to identify the presence of motion impairments and address it alongside symptomatic interventions like medication and injections. The regression cascade may occur when the initial intervention for regional joint pain is only symptomatic with continued demands in the presence of motion impairments. While this concept applies to both the spine and extremities, this study highlights this approach relevant to the extremities highlighting the most common motion impairments seen in mechanical dysfunction of the lower extremity. The most appropriate referral when a correlation between a painful joint and a motion impairment has been made would be to the physical rehabilitation professional capable of managing motion impairments. Lastly, it may be important to know that motion impairments have a collective presentation. An individual presenting with ankle pain may present with motion impairments in the ankle, hip, and knee. The takeaway here is for the clinician at a primary level to be vigilant about the need to investigate the presence of a distal ankle and foot motion impairment when examining an individual with knee or hip pain and vice versa. Lastly, outcome studies to support the validity of this claim that these preventive measures indeed do prevent the progression of mechanical musculoskeletal disorders are recommended.
